# Imaginative Thought

**DOI:** 10.5334/joc.506

**Published:** 2026-07-01

**Authors:** Ruth M. J. Byrne

**Affiliations:** 1 Trinity College Dublin, University of Dublin, Ireland

**Keywords:** Imagination, moral decision-making, false beliefs, causal reasoning, explanations, Artificial Intelligence, counterfactual conditionals, deductive inference

## Abstract

I suggest that the imagination is the engine of the mind. Its power can be illustrated in several domains that otherwise may seem quite disparate. I start first with moral decision making. Experimental results show the power of our imagination in bringing about immediate change in our moral judgments. Even actions we consider completely morally unacceptable can become neutralised. The impact of the moral imagination has many consequences, including its effects on our ability to understand other people’s perspectives and their sometimes false beliefs. Next, I turn to causal reasoning. Experimental results show that the entertainment of counterfactual possibilities drives our causal explanations, even though causal and counterfactual explanations can have very different content. The impact of the causal imagination has many consequences including its effects on our ability to understand decisions made by Artificial Intelligence decision support systems. In the final section, I consider the mechanics of the imagination and the cognitive processes that underlie the mental simulation of alternatives to reality. Experimental results show that we construct mental models to represent the meaning of a counterfactual conditional. Our imagination of multiple possibilities has many consequences, including its effects on counterfactual inferences.

## Introduction

One of the most remarkable aspects of the human mind is that we are not restricted to thinking only about facts, or about here and now, and instead we can imagine alternatives to reality, about the past, present and future. Our ability to do so is central to the creation of literature and poetry, film and theatre, painting and music; and it underpins scientific and medical discoveries, as well as inventions, design, and innovation. But our imagination is also central to much of our everyday thinking, evident in our engagement with pretense, daydreams, fantasies, and even our appreciation of fiction. It is prevalent in our mind-wandering and the enduring allure of “what if…”, and “if only…” thoughts. Imagined alternatives to reality entertain us, but they are no mere frivolous aside; they play a serious and central role in cognition. In this invited article, to mark the award of the European Society for Cognitive Psychology (ESCOP) Broadbent Prize and the presentation of the Broadbent Lecture at the ESCOP 2025 Conference, I suggest that the imagination is the engine of the mind. To make this case, I illustrate the power of the imagination in several domains that may otherwise seem quite disparate. I start first with moral decision-making, then I consider causal reasoning, and finally, I discuss the mechanics of simulation.

My illustration of the workings of the human imagination is made with reference to counterfactual imagination. We often imagine how things could have turned out differently “if only…”, particularly after a bad outcome (e.g., Roese & Epstude 2017; De Brigard & Parikh 2019). For example, suppose you fail an exam. You might imagine an alternative to the outcome, “I wouldn’t have failed the exam if only …” (Kahneman & Tversky 1982; Byrne 2005). You might think back about the events that occurred before the bad outcome, such as the amount of time you spent playing sports, or the long family holiday you went on, or the many occasions you had coffee with friends, and you might identify one of these events as crucial to the bad outcome, concluding, “I wouldn’t have failed the exam if only I hadn’t spent so much time socialising with my friends”. In this way, our counterfactual imagination can help us to detect the causes of outcomes, to explain events (see Byrne 2016 for a review). It may be a key learning mechanism that enables us to infer how to prevent bad outcomes from recurring. We can form intentions to avoid repeating past mistakes, for example, you might resolve, “next semester I won’t socialise so much” (e.g., Roese & Epstude 2017; Girotto et al. 2007; Markman et al. 2008a). And of course, you may experience regret when you imagine how things could have turned out differently. Emotions such as regret, remorse and guilt, or hope and relief, arise because we think about the way the situation turned out, and imagine how it could have turned out differently, and in the comparison of the two, the emotion arises or is amplified (e.g., Morrison & Roese 2011; Gilovich et al. 1998; Sweeny & Vohs 2012; Feeney et al. 2017). And thoughts about what could have been done differently, and should have been done differently, also underpin our moral judgments about blame and responsibility. You may believe it is your own fault that you failed the exam, and others may also attribute responsibility to you for the outcome. Or, you may blame your friends for distracting you from your studies. The effects of the imagination on moral decisions are particularly revealing about its key role in cognition. The first clues I wish to discuss about the power of the imagination as the engine of the mind come from evidence of its profound effects on our moral cognition.

## Moral Imagination

We make moral judgments often in our daily lives, including judgments of blame, responsibility, fault, accountability, and punishment. The dominant view of how we do so is that we apply firmly established rules about harm, fairness, purity, respect, and so on, that are based on our long-standing principles and values, acquired in childhood through our family, education, culture, and society. On this view, our moral judgments may often be very immediate and intuitive, with little role for reflective thought, other than for post-hoc justification (e.g., Graham & Haidt 2012); or perhaps they may depend on a combination of intuitive and reflective processes (e.g., Greene et al. 2001; for discussion see Gürçay & Baron 2017; Goodwin 2017; Gray & Schein 2012). As a result, our moral judgments may seem to be entrenched in cherished moral standards, and we may be reluctant to modify our long-held moral beliefs. Yet, as we will see, such is the power of our imagination that it can have an immediate impact to change our moral decisions quite dramatically.

### Moral decision making

How does our imagination change our moral judgments? To illustrate the impact of counterfactual imagination on moral cognition, consider how our moral judgments can shift, so that we can come to consider a morally unacceptable action to be acceptable (Tepe & Byrne 2022). Moral changes generally occur over the course of generations as social norms shift, so that matters once considered morally unacceptable may become acceptable, such as gay marriage, and matters once considered acceptable may become morally unacceptable, such as child labour. But changes in moral judgments can also occur on a much shorter timescale within our own minds, for example, when we learn new information and revise our beliefs. However, most of us tend to be resistant to changing our moral judgments even when presented with opposing reasons (e.g., Stanley et al. 2018; Skitka 2010; see also Monroe & Malle 2019; Sabo & Giner-Sorolla 2017). What then can bring you to moderate your moral views, so that something you believe is morally unacceptable can come to seem not so?

Consider the following example. Suppose you hear that a passenger in an airplane does not want to sit next to a Muslim passenger and so he tells the stewardess the passenger must be moved to another seat. How morally acceptable do you think that is? You will probably judge that it is not at all acceptable. We asked participants in several experiments to judge the moral acceptability of a set of immoral actions including this example. They indicated the extent they thought the behaviour was morally acceptable by providing their judgments on a scale with 0 labelled “not at all” and 100 labelled “definitely.” They generally tended to judge such actions to be very morally unacceptable, around 5–10 on the scale. Now consider, what would make you change your mind about your judgment? We discovered that participants changed their minds and judged such actions very differently after a mere few seconds of imagining circumstances in which they would be moral (Tepe & Byrne 2022). For each action we asked them to try to imagine some different circumstances in which the behaviour would be morally acceptable, writing their response by completing the sentence stem, “it would be morally acceptable if…”. If you were to do this task, you may think of various circumstances, as our participants did, such as that the Muslim passenger had been rude to the protagonist and so the reason he wanted the Muslim passenger to be moved wasn’t anything to do with religion. Or that the Muslim passenger had been acting suspiciously and the reason the protagonist wanted him to be moved was to protect other passengers. After participants imagined different circumstances in which the behaviour would be morally acceptable, we asked them to again provide their judgment about the extent to which they thought the behaviour was morally acceptable, given the circumstances they had just considered. They now made their judgments around the mid-point of the scale, about 45–50, in other words the moral unacceptability of the action was neutralised. Although at the outset participants had judged the action to be very morally unacceptable, their judgments changed substantially, just because they imagined some different circumstances in which it would be morally acceptable (Tepe & Byrne 2022).

The results show that participants’ moral judgments can change considerably, in the absence of any new facts, when they merely imagine some circumstances in which a morally unacceptable action would be moral. This moral shift did not occur when participants carried out a factual task such as providing a label for the behaviour. They could readily label the action, for example, they named it as racism, or religious intolerance. And when these participants were asked to provide their judgments again, they continued to judge the actions to be highly morally unacceptable. Hence, the moral shift that occurs when participants imagine alternative circumstances does not merely reflect repetition of the judgment task or other task demands. Instead, after they imagined alternative circumstances, participants seemed to interpret the situation to be quite a different one, even though they had been given no further facts, or any arguments to persuade them the action was justified. Imagining alternative circumstances provides people with remarkable moral flexibility, and allows circumstances to moderate their moral judgments.

Strikingly, the moral shift occurred whether participants were given as much time as they wanted to imagine circumstances in which an action would be morally acceptable, or whether they were placed under very limited time constraints. We gave some participants just 20 seconds to read about the behaviour and imagine circumstances in which it would be morally acceptable, a time we had pretested which enabled them only to quickly jot down a word or two to convey the first thought that came to mind. Even very switfly writing down a few words about how the action could be morally acceptable had a dramatic impact on their judgments of moral acceptability. Clearly, they could bring to mind circumstances in which the action would be moral, in just a matter of seconds. Alternative possibilities seem to be very rapidly accessible (Orenes et al. 2022; Phillips et al. 2019). Often we tend to imagine how things could be better rather than worse (e.g., De Brigard et al. 2013; Rim & Summerville 2014), and so we tend to change something morally bad in the actual world by imagining something better in its place. The experimental results suggest that the moral imagination does not appear to require much effortful reflection.

Moreover, the impact of immediate counterfactual thoughts were similar to more reflective ones, on participants’ judgments of the moral acceptability of the action. When participants were encouraged to take all the time they needed to imagine circumstances in which the action would be morally acceptable, their reflective thoughts revealed a range of counterfactual argumentation strategies (see Tepe & Byrne 2022 for details). In some cases, they re-interpreted the action by challenging the facts, to conjecture it was not immoral because other facts explained it, such as that the Muslim passenger had been rude, and so the protagonist’s action was not in response to religion. In other cases, they re-interpreted the action as a dilemma that acknowledged the action as immoral but assumed it was in conflict with another moral principle which had to over-ride it, such as that the Muslim passenger had been acting threateningly, and so the protagonist’s action was justified in the service of another moral principle, to protect others. Participants rarely tended to reject the task and say for example, that such an action is just never morally acceptable, or that only the opposite action is morally acceptable. They also rarely suggested that the action could be moral in another possible world with very different norms (see e.g., Gendler 2000).

Our imagination can lead us to change our minds about a situation we initially judged as morally unacceptable. We can excuse or defend, explain or explain-away, an otherwise immoral act by deploying our imagination to re-interpret it, as not what it seemed. And this moral mitigation can occur in the absence of any additional facts. The example highlights how our imagination may set our moral compass, or at least move it around its reference point (Knobe 2010). Moral judgments are of course influenced by many factors, such as our emotions (e.g., Greene et al. 2001; Haidt 2001; Gubbins & Byrne 2014), and our inferences about an actor’s intentions (e.g., Cushman 2008; Young & Saxe 2011; Parkinson & Byrne 2018). But our imagination of how things could have turned out differently has wide-spread effects that can amplify or decrease our judgements of blame, moral responsibility, and punishment (e.g., Branscombe et al. 1996; see also Alicke et al. 2008; Malle et al. 2014). The impact of our imagination is so great, that we blame someone who carries out a harmful action which did not result in a bad outcome, just as much as someone who carries out the same action with a bad outcome, once we can imagine how the action could have led to the bad outcome (e.g., Parkinson & Byrne 2017a; see also Lench et al. 2015). Similarly, we praise someone who carries out a helpful action which did not result in a good outcome, just as much as someone who carries out the same action with a good outcome, once we can imagine how the action could have led to the outcome (Byrne & Timmons 2018; see also Timmons et al. 2019). The moral imagination exerts tremendous influence over our judgments of good and bad. This impact has many implications, such as for understanding other people’s perspectives, to which we now turn.

### Imagination and other people’s perspectives

How does our imagination help us to understand other people’s perspectives? The effects emerge early, as children develop their counterfactual imagination during childhood, which in turn supports their ability to understand that other people’s beliefs may be different from their own (e.g., Beck et al. 2009; Riggs et al. 1998; Guajardo et al. 2009; Perner et al. 2004). It can be difficult to see things from another person’s perspective. Children begin to learn that others may see the world differently from them during their early years, but the process continues even into adulthood (e.g., Dumontheil et al. 2010). For example, it takes several years before children, around the age of 4 years, begin to understand that other people may have different beliefs about the world compared to their own beliefs, and to realise that other people may not know what the child him- or herself knows. Consider the well-known scenario in which Sally puts her ball in her basket and goes out for a walk. While Sally is away, Ann moves the ball to her box instead. When Sally comes back where will she look for her ball? Children by about the age of 4 years begin to respond reliably to such questions in ways that show they understand that Sally believes her ball is still in her basket, even though the child knows it is in Ann’s box (e.g., Wimmer & Perner 1983; see Wellman et al. 2001). Working out where Sally thinks her ball is requires you to make the inference that if Anne haven’t moved the ball, it would still be in the basket (e.g., Robinson & Beck 2000). Children’s answers to false belief questions such as “where does Sally think her ball is?”, and to counterfactual questions such as, “if Anne haven’t moved the ball, where would it be?” are highly correlated (e.g., Riggs et al. 1998; Müller et al. 2007; Perner et al. 2004). Their gradual understanding that others may have false beliefs potentially depends on the prior development of their ability to imagine how things could have been different. Once children can imagine how things could have been different, they are able to imagine a situation from different perspectives. They develop the ability to mentally represent an event, and the ability to understand that their simulation is a representation of what happened. They also develop the skills to simulate how an event could have turned out differently, and to understand that this counterfactual simulation provides a different perspective. These cognitive skills enable children to imagine how things might seem from another person’s perspective.

Of course, to answer a counterfactual question such as “if the dog hadn’t run across the kitchen floor with his dirty paws, would the floor still be clean?” requires representing the physical state of the world. In contrast, to answer a corresponding false-belief question such as, “will Abigail, who was in the other room when the dog ran across the kitchen floor with his dirty paws, think the floor is still clean?” requires instead understanding a person’s mental state (e.g., Guajardo et al. 2009; see also Miller 2009). Counterfactual reasoning may develop before false-belief reasoning simply because children can reason about physical states before they can reason about mental states. But we created a task to enable us to test false-belief reasoning about mental states and compare it to counterfactual reasoning about mental states. And we found that children’s counterfactual reasoning develops before false-belief reasoning even when both concern mental states. The new task we developed is analogous to the Sally-Anne unexpected change-of-locations task, but about an unexpected change-of-intentions (Rasga et al. 2016). It describes a change, not in the location of an object in the physical world, but in the internal reason a person has in their mind for carrying out an action. Consider, for example, Roy and Betty who are in their sitting room. Roy hears Betty say she wants to write a letter to send to her friend. Roy goes into the garden, and while he is away, Betty’s father tells Betty to do her homework. When Roy comes back into the living room, he sees Betty writing on paper. Suppose you are asked, what does Roy believe is the reason that Betty is writing on paper? When children are asked this false belief question, they need to understand that although they themselves know that Betty’s reason is to do her homework, Roy will think she still has her original reason, to write a letter to her friend. They need to keep in mind that they know, in reality, Betty is writing on paper to do her homework, but in Roy’s mind, Betty is writing on paper to write a letter to send to her friend. Suppose now you are asked, If Betty’s father hadn’t told Betty to do her homework, what would have been the reason that Betty was writing on paper? When children are asked this counterfactual question, they need to consider the situation as they know it is currently, Betty’s reason for writing on paper is because her father told her to do her homework, and they need to also consider the situation as it once was but is so no longer, Betty’s reason for writing on paper was because she wanted to write a letter to her friend. They need to keep in mind that they know, in reality, Betty is writing on paper to do her homework, but that in the conjectured alternative to reality, Betty would be writing on paper to write a letter to send to her friend.

Both questions require children to think about mental states in the same situation, Betty’s reasons for her action. We found that only about one-third of typically developing children at the age of 6 years were able to answer both questions correctly. About one-third of them gave the incorrect answer to both instead. And for the remaining one-third, they tended to be correct on the counterfactual inference but incorrect on the false-belief one. We rarely encountered children who could make the false belief inference but not the counterfactual one. In contrast, by 8 years of age, most of the children made the correct inference for both questions. The results showed that even when counterfactual and false-belief inferences are both about mental states, there is still a strong correlation between them. The accuracy of the false belief inference appears to depend on the accuracy of the counterfactual inference.

Interestingly, very similar results occur for children with autism. Although the development of accuracy in both sorts of inferences is somewhat delayed for autistic children, we observed very similar patterns (Rasga et al. 2017). The results are consistent with the observation of enhanced reasoning and decision making in autistic individuals in some cases (Morsanyi & Byrne 2019). Overall, the results about false belief reasoning and counterfactual reasoning in children provide strong support for the proposal that imagining how things could have been different forms the basis for our ability to imagine other people’s perspectives.

The development of simulation skills to imagine different perspectives is no mean feat. To create an alternative to reality, children first need to be able to mentally represent the facts of a situation, e.g., Betty wanted to write a letter. Then they must be able to modify their mental representation, by deleting some of the information about what happened, say, Betty does not want to write a letter, or by adding some new information, say, Betty wants to do her homework. Their comparison of their simulation of an alternative to reality, to their representation of reality requires working memory capacity to keep in mind at least these two possibilities. It also requires inhibitory skills to suppress their attention to one possibility, the representation of reality, so that they can focus on the imagined alternative. And it requires attention-switching skills to attend to one possibility or the other to update each one (e.g., Drayton et al. 2011; Robinson & Beck 2000; Müller et al. 2007; Feeney et al. 2017). Perhaps unsurprisingly, the ability to compare reality to an alternative continues to develop throughout middle childhood (e.g., Beck et al. 2006; Rafetseder et al. 2013). Counterfactual thoughts may help children to understand that their mental simulations are representations of the world, and that they must keep track of what is real, and what is imagined (Espino & Byrne 2021; Perner et al. 2004).

As these examples illustrate, the imagination has substantial effects on moral and social understanding and inference. I turn now to a domain that may appear at first to be quite disparate from moral and social cognition, but one which shows very similar effects of the imagination, that of causal cognition.

## Causal Imagination

We make causal judgments often in our daily lives, including inferences about causes and their effects, and inferences to identify causal responsibility in situations for which there are multiple potential causes. Once again, the dominant view of how we do so is that we apply well-established rules, based on principles embedded in our causal models of the world, and encapsulated in probability distributions that capture our degrees of belief and certainty (e.g., Pearl 2013; Sloman & Lagnado 2015; Meder et al. 2010). On this view, we make causal inferences by computing Bayesian probability calculations to assess the nature of relationships, assessing strengths of associations and in some cases computing interventions to interrogate the effects of the absence of causes or outcomes. Yet, here too our imagination has a profound and nuanced impact on our causal cognition, as we will now see.

### Counterfactual and causal explanations

The idea that we understand that “A caused B” by thinking that “if A hadn’t happened then B wouldn’t have happened” has a long history in philosophical analyses of causation (Hume 1739; Mill 1843; Lewis 1973; Mackie 1974; Stalnaker 1968; Williamson 2018). Causal inference and counterfactual imagination have often been considered as two sides of a single coin. And there are certainly close links between the counterfactual alternatives we create and our causal ascriptions in many domains (e.g., Hart & Honore 1959; Einhorn & Hogarth 1986; McGill & Klein 1993). When we can imagine that a different outcome would have occurred if an event preceding the outcome hadn’t happened, our judgments that the preceding event caused the outcome increase (e.g., McEleney & Byrne 2006; Henne et al. 2019). Consider a runner who sustains a minor ankle injury in training and then loses her race by milliseconds when she experiences side effects of fatigue after taking a legal painkiller. Suppose there was another painkiller available that did not cause side effects. Participants judged that the painkiller’s side effects caused the runner to lose her race. They were inclined to consider the painkiller had a strong causal role when they could readily imagine a counterfactual alternative, “if only she had taken the other painkiller” (McCloy & Byrne 2002; see also N’gbala & Branscombe 1995). And just as their causal judgments were amplified when they knew about a counterfactual alternative, such as a painkiller with no side effects, so too their causal judgments were diminished when they knew about a “semi-factual” alternative, that is, a different antecedent that led to the same outcome, such as another painkiller with the same side effects. They were inclined to judge that the painkiller did not have a strong causal role when they could readily imagine a semi-factual alternative, such as “even if she had taken the other painkiller, she still would have experienced the same side effects”. When we try to work out the causes of events, we are influenced by such available possibilities (e.g., Gerstenberg et al. 2017; 2021; Johnson-Laird et al. 2023; Kominsky et al. 2015; Icard et al. 2017; Lagnado et al. 2013; Quillien & Lucas 2023).

To illustrate just how powerful our imagination can be, consider its role in our identification of a cause from a set of potential causes. Although we sometimes rely on our imagination to help us figure out what caused an outcome, we also do so to excuse past mistakes, to explain-away an outcome, or to justify a decision, e.g., “if only I’d known sooner…” (Catellani & Covelli 2013; Brockbank et al. 2024; Markman et al. 2008b; Walsh & Byrne 2007). Are the causes we identify in our counterfactual explanations the same as those in our causal explanations? There are of course, important differences between counterfactual thoughts and causal explanations. One difference is that we tend to construct causal explanations more readily than counterfactual ones (McEleney & Byrne 2006). When participants were asked to write a diary entry about a fictional set of events, unprompted by instructions to think counterfactually or causally, they spontaneously created causal explanations, such as “I didn’t make new friends because I didn’t go to my neighbour’s party”; an explanation which focuses on the facts of the matter, not going to the party and not making new friends. They constructed far more of these causal explanations than counterfactual ones, such as “I would have made new friends if I had gone to my neighbour’s party”; an explanation which focuses not only on the facts but also on potential alternatives, going to the party and making new friends (McEleney & Byrne 2006).

Perhaps surprisingly, another key difference is that counterfactual and causal explanations sometimes differ in their content. Consider a car crash that happened when a drunk driver swerved into a protagonist who was driving home from work on a route he didn’t usually take. Participants tended to imagine that the accident wouldn’t have happened if the protagonist had driven home by his regular route; but they tended to say the cause of the crash was the drunk driver (Mandel & Lehman 1996; N’gbala & Branscombe 1995; Deighan & Byrne 2025). The difference might be considered puzzling given the proposal that causes are identified by considering counterfactual possibilities. It could even be taken to support the alternative causal pluralist claim that only some causes are identified by inferences about whether they make a counterfactual difference to the outcome, say the protagonist’s route home; whereas other causes are instead perceived in terms of the transference of energy by mechanisms or forces, say the drunk driver swerving into the protagonist (e.g., Dinh & Danks 2021; Godfrey-Smith 2010; see also Wolff 2007; Wolff & Thorstad 2017; Walsh & Sloman 2011).

However, the puzzle about the difference in the sorts of causes identified in counterfactual and causal explanations is resolved when we consider that both rely on counterfactual possibilities, but avail of different cues to select a cause from the set of possibilities. For example, counterfactual thoughts tend to focus on events within a protagonist’s control. Suppose you are told that your friend could win some chocolates if she does a multiplication sum in her head in 30 seconds. The sum will be either an easy one with two one-digit numbers (e.g., 7 × 3) or a difficult one with two two-digit numbers (e.g., 67 × 83). The sums are in two sealed envelopes, marked A and B. Your friend decides to try it and chooses envelope A; unfortunately, it contains the two-digit sum. She tries to multiply them in her head in 30 seconds but she can’t, and so she doesn’t win any chocolates. When participants imagined how the protagonist might think about how things could have turned out differently, they tended to say, “if only she had chosen the other envelope”. In other words, they tended to refer to the decision within her control rather than the many aspects of the situation outside her control, such as the time limit, the lack of pen and paper, the need to concentrate, and so on (Girotto et al. 2007; see also Ferrante et al. 2013; Pighin et al. 2011; Mercier et al. 2017). To examine counterfactual explanations in this situation, we asked participants to suppose that afterwards, your friend tries to give the best explanation she can for what happened, and she begins, “it wouldn’t have happened if…”. Even when participants try to provide their best explanation, they tended to say “if she had chosen the other envelope”, that is, they focused more often on the event within her control, than on events outside her control. But we also asked them to suppose that afterwards, your friend tries to give the best explanation she can for what happened, and she begins, “it happened because…”. This time they provided causal explanations such as, “because she had just 30 seconds”, “because she was not allowed to use a pen and paper”, “because she could not concentrate”, “because she wasn’t good at mathematics”, or even “because she had bad luck”. In other words, they identified aspects of the situation that were outside her control, as often as ones that were within her control such as the envelope choice (Deighan & Byrne 2025).

Why does the difference in content occur? Participants simulate a set of possibilities that includes the factual events, e.g., the several causes described in the story, the envelopes containing the simple one-digit sum and the difficult two-digit sum, the time limit, the requirement to compute the sum in her head, and they simulate their outcome, she didn’t solve the sum. In addition, they simulate various counterfactual alternatives in which the outcome is different and she won the chocolates, if she had had longer, if she had had pen and paper, if she had chosen the other envelope. When they create a causal explanation, their emphasis is on explaining what happened and they focus on strong causes that covary with the outcome, e.g., she didn’t solve the sum because she didn’t have enough time. Their choice from among the set of possibilities may be guided by considerations of simplicity, likelihood, or breadth (e.g., Khemlani et al. 2011; Liquin & Lombrozo 2020; Liefgreen & Lagnado 2023; Johnson et al. 2019; Zemla et al. 2017). But when they create a counterfactual explanation, their emphasis is on explaining what could have happened instead and they focus on the cause that can be most easily modified in the construction of their counterfactual simulations, the enabling background condition for the outcome, if she had chosen the other envelope she would solved the sum. Their choice from among the set of possibilities is guided by considerations such as the availability of alternatives. For some causes, an alternative can be readily inserted as a substitute, e.g., envelope A can be replaced by the readily available alternative of envelope B. The mutability of an event reflects the availability of an alternative, including the ease with which the alternative comes to mind. The ready availability of an alternative possibility can be determined by various factors such as norms or defaults (e.g., Kahneman & Miller 1986; Feldman et al. 2020; see also Phillips et al. 2019). Our counterfactual explanations are guided not by cues such as comprehensiveness or simplicity but instead by factors such as controllability or exceptionality that reflect the availability of alternative possibilities.

Remarkably, we all tend to create very similar sorts of counterfactual alternatives to reality. We tend to change exceptional actions to be normal (e.g., Kahneman & Tversky 1982; Dixon & Byrne 2011), and controllable events more than uncontrollable ones (Girotto et al. 1991; Markman et al. 1995; McCloy & Byrne 2000). And we also often change actions more than inactions, for example, if someone loses money when she switched her shares to a different company, we judge that she will feel more regret than someone who loses money when she decided to keep her shares in the same company (e.g., Byrne & McEleney 2000; Kahneman & Tversky 1982; see also Gilovich & Medvec 1995). We tend to change first causes in a causal sequence (e.g., Segura et al. 2002; Wells et al. 1987), but more recent events in a temporal non-causal sequence (e.g., Miller & Gunasegaram 1990; Walsh & Byrne 2004). These factors seem to correspond to pivotal junctures in our representation of reality, “fault-lines” of our imagination, that guide our creation of counterfactual possibilities (Kahneman & Tversky, 1982). They have a knock-on effect for the sorts of counterfactual explanations we create, determining the causes we choose from the counterfactual possibilities we consider. As a result, counterfactual and causal explanations can have very different content even though they both arise from the consideration of counterfactual possibilities. In such ways, our causal imagination exerts tremendous influences over our judgments of cause and effect. This impact has many implications, including for example, for understanding decisions made by Artificial Intelligence (AI) decision support systems, to which I now turn.

### Counterfactual explanations in eXplainable AI

The nature of counterfactual explanations has recently come under close scrutiny, in part because of the remarkable proliferation of algorithms in eXplainable AI (XAI) that generate automated counterfactual explanations. AI decision support systems are becoming more commonly used in our everyday lives, ranging from education to commerce, from health decisions to legal judgments, from financial decisions to travel advice. Yet many of us have concerns about their fairness and transparency and may sometimes be reluctant to trust their decisions (e.g., Binns et al. 2018; Dodge et al. 2019). Our willingness to accept AI decisions is influenced by many factors, including the provision of an explanation (e.g., Liao & Varshney 2021; Hoffman et al. 2018; Verma et al. 2020; van der Waa et al. 2021). And recently, counterfactual explanations have been proposed to be particularly suitable for explaining AI decisions (for reviews see Karimi et al. 2022; Keane et al. 2021). They describe how an AI system’s output decision would have been different, if some of the input features had been different, for example, a bank customer who has been refused a loan by an automated system may be provided with the counterfactual explanation, “if you had asked for $2k less, you would have received the loan.” Strikingly, more than several hundred distinct algorithms designed to generate automated counterfactual explanations are now available (e.g., Keane et al. 2021; see also Byrne, 2019; Miller, 2019).

Do counterfactual explanations help us to understand AI decisions? Recent studies show that participants do indeed feel that counterfactual explanations help them to understand an AI’s decisions, as indicated by their self-reports on subjective measures such as ratings of whether they considered an explanation to be helpful (e.g., Lim et al. 2009; Lucic et al. 2020; van der Waa et al. 2021; Lage et al. 2019). Indeed, their subjective ratings of the helpfulness of counterfactual explanations tend to be higher than their ratings of other sorts of explanations, such as matched causal explanations (Celar & Byrne 2023; Warren et al. 2024). But objective measures tell a somewhat different story. Consider a situation in which participants gain experience with an AI decision support system by observing its decisions for many different instances and receiving an explanation for each decision. Then they are presented with a variety of new instances that the AI system must make decisions about, and participants are asked to predict its decisions. Their accuracy in predicting the AI system’s decisions provides an objective measure of whether the earlier explanations really did help them to understand the AI system’s decisions. Studies of this sort show that counterfactual explanations do indeed improve participants’ accuracy in predicting an AI system’s decisions compared to no explanation. But such studies also show that other sorts of explanations, such as causal explanations, improve participants’ accuracy just as much (e.g., Celar & Byrne 2023; Warren et al. 2024). Counterfactual explanations do not appear to enjoy significant advantage over other sorts of explanations (e.g., Lim et al. 2009; Lucic et al. 2020; van der Waa et al. 2021; Lage et al. 2019).

A related question is, do counterfactual explanations persuade people to switch from their own preferred decision to one proposed by an AI system? To find out, we gave participants a well-known problem for which most of us have extremely robust decision preferences (from Tversky & Kahneman, 1981). Imagine the US is preparing for an outbreak of an unusual disease which is expected to kill 600 people. Two alternative programs to combat the disease have been proposed. Assume the consequences of the programs are as follows: If program A is adopted, 200 people will be saved. If program B is adopted, there is a 1/3rd probability that 600 people will be saved, and a 2/3rd probability that no-one will be saved. Which of the two programs would you choose? When the options are framed in this way in terms of lives that could be saved, most of us choose the certain option: program A (Tversky & Kahneman, 1981). In contrast, most of us choose the risky option: program D, when the options are framed instead in terms of lives that could be lost, i.e., if program C is adopted, 400 people will die; If program D is adopted, there is a 1/3rd probability that no-one will die, and a 2/3rd probability that 600 people will die. Our decision preferences are so robust that we tend to blame another person far more harshly for a bad outcome when they have not made the typical decision we would have made (Parkinson & Byrne, 2017b). Such compelling preferences allow a strong test of whether people are willing to accept an AI system’s proposal of the opposite decision. For example, suppose you make the typical decision of the certain option A in the lives saved version, but you are then told that an AI system proposes the opposite decision, program B. Will you stick with your original certain choice, or switch to the AI system’s proposed risky decision instead? Or suppose you make the typical decision of the risky option D in the lives lost version, but you are then told that an AI system proposes the opposite decision, program C. Will you stick with your original risky choice, or switch to the AI system’s proposed certain choice instead? We found that participants who made the typical decision tended to be very reluctant to switch to an AI system’s proposed opposite decision, only about one-fifth to one-quarter of participants did so in our series of experiments (Dai et al. 2026). By comparison, when a human expert proposed the opposite decision, about half of participants switched.

But a counterfactual explanation made a difference. Suppose the AI system recommends program B, and it says that in previous cases the outcome would have been worse if program A had been chosen. We found that the explanation was effective in persuading some participants to switch from their decision to the AI system’s one: about one-third to nearly one-half of participants did so in our series of experiments. They tended to be persuaded by a counterfactual whether it was about how things could have turned out worse, or about how things could have turned out better. Intriguingly, the corresponding causal explanation, i.e., the AI system recommends program B, and it says that in previous cases the outcome was good because Program B was chosen, did not seem to have such an effect (Dai et al. 2026). Why would a counterfactual explanation be effective in persuading people to consider a different decision? Our preferences seem to arise, at least in part, because we have not explicitly simulated all possible aspects of the situation. For example, in the unusual disease problem, we may tend to immediately represent only the gist of the available choices (e.g., Reyna et al. 2013; Gonzalez et al. 2005; see also De Martino et al. 2006; Kahneman & Frederick 2007). When we understand that option A will lead to 200 of the 600 lives being saved, we do not seem to have thought through the “opposite side of the coin”, that it also means that 400 of the 600 lives will be lost. Counterfactuals may help us to think through alternative possibilities, and ensure that we mentally represent relevant aspects not only of what will happen, but also of what could happen. The example shows that imagining a counterfactual alternative to what happened can have a profound influence, in this case on how we consider decisions of an AI decision support system.

As these illustrations show, the imagination has substantial effects on causal inference and explanation. I turn now to consider the mechanics of the imagination, the cognitive processes that underlie our mental simulation of alternatives to reality.

## Simulation

How do we imagine possibilities? In this final section I consider the nature of the mental simulations that support the imagination of possibilities. I sketch the cognitive processes that enable us to construct models of multiple possibilities, and their effects on the counterfactual inferences we can make. Once again, a prominent view of how we do so is that we apply established rules, based on formal logics, or on Bayesian probability (e.g., Rips 1994; Oaksford & Chater 2007; Evans & Over 2004; see Knauff & Gazzo Casteneda 2023 for a review). Yet, once again our imagination has a dramatic impact on our reasoning, to which we now turn.

### Counterfactuals and possibilities

Suppose you hear a conjecture, such as “if there had been roses in the shop then there would have been lilies. You will immediately imagine not only what was mentioned in the conjecture, the roses and lilies, but you will also begin to think about what must be the facts, that there are no roses and no lilies. We gain access to both situations very rapidly (e.g., Orenes et al. 2022; Ferguson & Jayes 2018; de Vega & Urrutia 2007; see also Kulakova & Nieuwland 2016). For example, participants listened to short descriptions such as a story about Michael going to a flower shop with his sister, and she told him that if there had been roses in the shop then there would have been lilies. As they heard the story, participants saw four images on a computer screen, one in each of its four quadrants, comprising an image of roses and lilies, an image of roses and lilies with a cross through it, an image of carnations and poppies, and an image of carnations and poppies with a cross through it. Eye-tracking cameras recorded where their eyes moved every 50 milliseconds. The results showed that at the outset of the description, most participants tended to look at the image of the roses and lilies, and the image of the carnations and poppies. But after they heard the first part of the counterfactual conditional, “if there had been roses…”, their eyes tended to stay on the image of the roses and lilies. And then as they heard the rest of the counterfactual, “…there would have been lilies”, their eyes tended to move to look at the crossed-out image of roses and lilies. The move to look at the crossed-out image of roses and lilies occurred very rapidly, within just a few hundred milliseconds. And for the remaining few seconds as they heard the counterfactual, their gaze alternated between the two images, the one of roses and lilies, and the one of crossed-out roses and lilies. The results show how our understanding of the multiple possibilities conveyed by a counterfactual conditional is remarkably rapid. We very swiftly simulate not only the situation corresponding to the imagined conjecture, but also the situation corresponding to the presumed facts. By comparison, when participants heard instead a description that contained a conditional only about the facts, “if there are roses then there are lilies”, they tended to look only at the image of roses and lilies (Orenes et al. 2019). When we understand a counterfactual conjecture, we are primed to try to recover the facts, to anchor our imagination of the conjecture in our understanding of the corresponding reality. Of course, sometimes we are swept up by our imagination into what almost happened, for example when a truck nearly knocks us down, our simulation of what could have occurred to us can seem overwhelmingly realistic. And at our other times our feet remain firmly on the ground, focused for example, on the facts that the truck did not touch us and nothing actually happened (e.g., Markman & McMullen 2003). The difference seems to reflect our ability to prioritise one simulation over another, our simulation of the imagined conjecture or our simulation of the corresponding facts, to keep track of the epistemic status of each one, and to switch our attention between them (e.g., Espino & Byrne, 2021).

What sorts of cognitive processes underpin our ability to construct models of multiple possibilities? Consider as a starting point, the cognitive processes that construct mental models corresponding to a factual description such as “either there is a triangle on the blackboard or else there is a circle”. The intuitive models that we immediately construct allow us to simulate that it is possible there is a triangle on the blackboard, and it is possible there is a circle on the blackboard (Khemlani et al. 2018; Johnson-Laird et al. 2023). Our initial models can be fleshed out when we think further about these possibilities. On deliberative reflection, we can make explicit in our model of the triangle on the blackboard that there is no circle, and in our model of the circle on the blackboard that there is no triangle. And when we subsequently observe that there is in fact a triangle on the blackboard, we can update our knowledge so that our model of the triangle now corresponds to a model of the facts rather than to a possibility (Khemlani et al 2018; Johnson-Laird et al. 2023). In this case, our model of a circle on the blackboard is now updated to a counterfactual possibility, which once was possible but is so no longer.

Similarly when we understand a conditional about facts, such as, “if there was an orange in the fruit bowl there was banana” our intuitive models simulate at the outset that it is possible there is an orange and a banana in the fruit bowl, and although we may be aware that there may be other possibilities, we may not flesh them out explicitly at the outset (Johnson-Laird & Byrne 2002). If we reflect further, we can simulate that it is also possible that there is no orange and no banana in the fruit bowl, and we may even simulate that it is also possible there is no orange but there is a banana in the fruit bowl. We can come to many different interpretations of a conditional in this way, and so our understanding of a conditional departs from the standard “material implication” interpretation prescribed by formal logic (Johnson-Laird & Byrne 1991). Our knowledge modulates the different combinations of possibilities that we may consider consistent with a conditional, leading to many distinct interpretations (Johnson-Laird & Byrne 2002). Strikingly, when we understand a counterfactual conditional such as, “if there had been an orange in the fruit bowl there would have been a banana”, we think about multiple possibilities from the outset. We think about the conjecture that there is an orange and a banana in the fruit bowl, and we keep track of the epistemic status of this possibility as corresponding to a counterfactual possibility. And from the outset we also think about the possibility that there is no orange and no banana in the fruit bowl, and we keep track of the epistemic status of this possibility as corresponding to the presupposed facts (Espino & Byrne 2021; Byrne & Johnson-Laird 2020). From the outset we construct multiple models to mentally represent the meaning of a counterfactual conditional. The dual possibilities we imagine ensure that we make very different inferences from counterfactuals compared to factual assertions, as we will now see.

### Counterfactual inferences

How do we make inferences about what didn’t happen, but once could have happened? Consider a counterfactual conditional such as “If Abi had pressed the button the machine would have stopped”. When you understand the assertion, you construct a model corresponding to the conjecture that Abi pressed the button and the machine stopped, and a model corresponding to the presumed facts, that Abi didn’t press the button and the machine didn’t stop. When you then hear that the machine did not stop, you can readily make the *modus tollens* inference, that Abi didn’t press the button. Participants make far more of such inferences from a counterfactual conditional, compared to the corresponding factual one, “If Abi pressed the button the machine stopped”. For the factual conditional, you understand it by constructing just a single explicit model corresponding to the possibility that Abi pressed the button and the machine stopped. You may not deliberate further to think of other possibilities at the outset, such as the possibility that Abi did not press the button and the machine did not stop. As a result, when you hear that the machine did not stop, you may not be so willing to make the *modus tollens* inference, that Abi did not press the button, because you have not yet thought about whether this possibility is consistent with the assertion. Participants make many more inferences such as *modus tollens* from counterfactuals compared to factual conditionals (Byrne & Tasso, 1999; see also Espino & Byrne 2020; Moreno-Rios et al. 2008). The result illustrates the role of the imagination in reasoning and corroborates the proposal that when people understand a counterfactual they simulate multiple possibilities from the outset. It is more difficult to explain if people instead follow fixed rules to calculate conditional probabilities of their beliefs or carry out interventions on their causal models (Evans & Over 2004; Oaksford & Chater 2007; Sloman & Lagnado 2015).

The impact of simulating multiple models for a counterfactual extends to other sorts of inferences. For example, when you hear “If Abi had pressed the button the machine would have stopped”, you will tend to infer, even without any further information, that the machine didn’t stop (Egan & Byrne 2012). You will tend to judge that it is highly probable that Abi didn’t press the button and the machine didn’t stop (Byrne & Johnson-Laird 2020). You will even be able to read “Abi didn’t press the button and the machine didn’t stop” very rapidly when it comes after the counterfactual conditional (Santamaria et al 2005; see also Frosch & Byrne 2012). Such demonstrations corroborate the idea that the cognitive processes that support our counterfactual imagination construct models of possibilities and keep track of their epistemic status, as corresponding to a counterfactual possibility or the facts.

We rely on our knowledge to construct models of possibilities (Byrne & Johnson-Laird 2020; Khemlani et al. 2018). For example, when you learn that “If Bill is in Rio de Janeiro then he is in Brazil” and you discover that he is not in Brazil, you can readily make the *modus tollens* inference that he cannot be in Rio, because you know that Rio is in Brazil (Johnson-Laird & Byrne 2002). In contrast, when you learn that “If Bill is in Brazil then he is not in Rio de Janeiro” and you discover that he is in Rio de Janeiro, now you will resist the *modus tollens* inference that he is not in Brazil, again because of your knowledge that Rio is in Brazil. Participants made twice as many of the *modus tollens* inferences from the former sort of premises compared to the latter (Johnson-Laird & Byrne 2002). Even when we imagine impossible things, such as, “if people were made of steel, they would not bruise easily”, we imagine them just as we do real possibilities, by trying to construct a consistent simulation of the impossible conjecture, informed by our knowledge. As a result we consider that some impossibilities could be true, and others could be false, such as “if people were made of steel they would bruise easily” (Byrne 2024).

As these illustrations show, the simulation of possibilities lies at the heart of our imagination, and it has wide-ranging effects on our reasoning.

## Conclusions

Possibilities are the foundational building blocks of thought, and our imagination of possibilities drives our cognition. The far-reaching influence of our counterfactual imagination is evident in this brief overview of its impact in several otherwise seemingly disparate domains. In the realm of moral cognition, the influence of our moral imagination is such that it can bring about immediate change to our moral judgments, so that even completely morally unacceptable actions can become neutralised. Its impact is evident from an early age, even on our ability to understand other people’s perspectives and their false beliefs. In the realm of causal cognition, the influence of our causal imagination is such that it can lead our causal and counterfactual explanations to have very different content. Its impact is evident even in our ability to understand decisions made by AI systems. The mechanics of the imagination rest on cognitive processes that simulate models of alternatives to reality. Our simulation of multiple possibilities of different epistemic status impacts our understanding and inference widely. As these examples aim to demonstrate, our imagination is the engine of our minds. Our remarkable ability to go beyond thoughts of facts, of the here and now, to imagine instead alternatives to reality about the past, present and future, is central to all of our cognition.
